# Cancer associated fibroblasts sculpt tumour microenvironment by recruiting monocytes and inducing immunosuppressive PD-1^+^ TAMs

**DOI:** 10.1038/s41598-019-39553-z

**Published:** 2019-02-28

**Authors:** Betul Gok Yavuz, Gurcan Gunaydin, M. Emre Gedik, Kemal Kosemehmetoglu, Derya Karakoc, Figen Ozgur, Dicle Guc

**Affiliations:** 1 Department of Basic Oncology, Hacettepe University Cancer Institute, Sihhiye, 06100 Ankara, Turkey; 2 Department of Pathology, Hacettepe University School of Medicine, Sihhiye, 06100 Ankara, Turkey; 3 Department of General Surgery, Hacettepe University School of Medicine, Sihhiye, 06100 Ankara, Turkey; 4 Department of Plastic Reconstructive and Aesthetic Surgery, Hacettepe University School of Medicine, Sihhiye, 06100 Ankara, Turkey

## Abstract

Fibroblasts turn into cancer associated fibroblasts (CAFs) in the tumour microenvironment. CAFs have recently attracted attention for their function as a regulator of immune cell recruitment and function in addition to their tumour-promoting roles. In this study, we aimed to determine the role of CAFs on monocyte recruitment and macrophage polarization in breast cancer. CAFs, which were α-SMA expressing fibroblasts in contrast to normal fibroblasts (NFs), effectively recruited monocytes. Recruitment of monocytes by CAFs might be mediated by monocyte chemotactic protein-1 (MCP-1) as well as stromal cell-derived factor-1 (SDF-1) cytokines. CAFs differentiated the recruited monocytes into M2-like macrophages which are capable of exerting their immunosuppressive roles via the PD-1 axis. CAF-educated monocytes exhibited strong immune suppression unlike NF-educated monocytes and enhanced the motility/invasion of breast cancer cells in addition to increasing the expressions of epithelial–mesenchymal transition (EMT)-related genes and vimentin protein in cancer cells. CAF-educated M1 macrophages displayed increased expression of M2 markers and production of anti-inflammatory cytokine IL-10 in contrast to decreased production of pro-inflammatory cytokine IL-12 compared with control M1 macrophages; suggesting that CAFs were also able to induce the trans-differentiation of M1 macrophages to M2 macrophages. We then investigated the relationship between the infiltration of CAFs and tumour associated macrophages (TAMs) using tissue samples obtained from breast cancer patients. High grade of CAFs significantly correlated with the number of TAMs in human breast cancer tissue samples. It was also associated with higher Ki-67 proliferation index, and higher tumour volume. This result is in line with our finding of increased breast cancer cell proliferation due to the effects of CAF-educated monocytes *in vitro*. Our results concluded that CAFs play pivotal roles in sculpturing the tumour microenvironment in breast cancer, and therapeutic strategies to reverse the CAF-mediated immunosuppressive microenvironment should be taken into consideration.

## Introduction

Tumours of epithelial origin grow in a complex and dynamic stroma consisting of stromal cells, immune cells, matrix proteins and soluble factors. This microenvironment provides all the necessary stimuli for tumour survival, growth, and invasiveness. Inflammatory cells and the pro-inflammatory cytokines they produce are essential components of this microenvironment^1^.

Monocytes originating from blood differentiate into either M1 or M2 subtype macrophages, depending on the environmental stimuli they receive^2,3^. Human M1 macrophages demonstrate elevated expressions of CD14, CD16, CD64, CD86, and Human Leukocyte Antigen (HLA)-DRα^3,4^; whereas, CD163 and CD206 are markers associated with M2 macrophages^5,6^. In breast cancer, macrophages represent up to 50% of the tumour mass^7^ and there exists a correlation between the number of tumour associated macrophages (TAMs) and poor prognosis^8^. Macrophages have roles in almost all stages of tumour progression^9^. In the primary tumour, they stimulate angiogenesis and facilitate invasion. During metastasis, macrophages prepare the pre-metastatic site and promote tumour cell dissemination as well as growth. They also generate an immunosuppressive microenvironment by disrupting natural killer (NK) and T cell functions^9^. TAMs usually resemble M2 macrophages^10^. During tumour development, tumour-infiltrating M1-polarized macrophages are generally identified by an IL-12^high^ IL-10^low^ phenotype and they enhance immune reactions that display anti-tumour effects. During late-stages of tumour progression, TAMs generally skew to an M2-like phenotype that is characterized by an IL-12^low^ IL-10^high^ phenotype and diminished tumouricidal activity^11^. The identification of molecules driving macrophage plasticity in the cancer microenvironment could provide a basis for macrophage-focused diagnostic and therapeutic strategies^12^.

Fibroblasts are one of the most abundant cell types found in the tumour stroma. Fibroblasts turn into cancer associated fibroblasts (CAFs) in the tumour microenvironment. As tumours are described as “wounds that never heal”^13^, CAFs are very similar to myofibroblasts, which are spindle shaped activated fibroblasts^14^. Although there is no specific marker that is exclusively expressed by CAFs, alpha smooth muscle actin (α-SMA) is widely used as a CAF marker^15^. CAFs not only play an active role in tumour initiation and progression, but also sculpt the tumour microenvironment^16,17^. CAFs have recently attracted attention for their function as regulators of immune cell recruitment as well as functions. Studies in breast cancer investigating the effects of stromal fibroblasts on monocytes/macrophages are very limited. In this study, we report for the first time that CAFs obtained from invasive breast cancer differentiated monocytes to M2-like pro-tumoural macrophages in terms of both phenotypic features and functions, in contrast to fibroblasts obtained from normal breast. We also demonstrated that CAFs were very effective in recruiting monocytes. Monocyte chemotactic protein-1 (MCP-1) and stromal cell-derived factor-1 (SDF-1) were found to be crucial monocyte chemotactic cytokines that were secreted from stromal cells.

## Results

### CAFs, which are α-SMA expressing fibroblasts in contrast to NFs, effectively recruit monocytes

**Figure 1:**
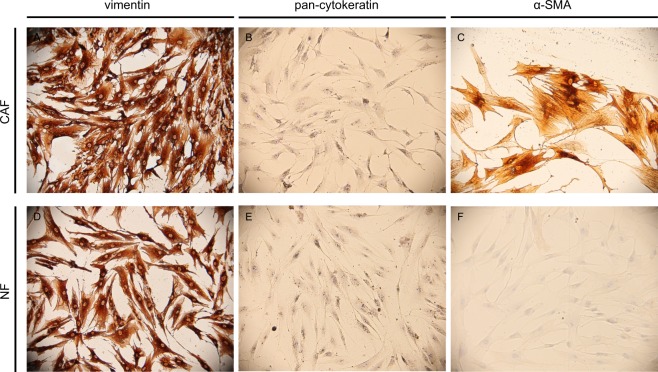
CAFs are α-SMA expressing fibroblasts in contrast to NFs. CAFs and NFs were cultured in 8-well chamber slides, then stained for vimentin (**A**,**D**), pan-cytokeratin (**B**,**E**) and α-SMA (**C**,**F**). Both CAF and NF cells were positive for vimentin and negative for pan-cytokeratin. However, CAFs were positive for α-SMA, unlike NFs. Sup. Figure 1 displays immunocytochemical stainings for fibroblast activation protein (FAP). One representative figure for each cell type is shown (×100).

CAFs were isolated from breast cancer tissues obtained from patients undergoing mastectomy. As healthy counterparts, we used normal fibroblasts (NFs) isolated from normal breast tissues obtained from patients undergoing reduction mammoplasty. Primary cultures of both of those isolated fibroblasts were established. All fibroblasts utilized in the experiments were characterized by vimentin, pan-cytokeratin and α-SMA expressions by immunocytochemistry. They differed in their expressions of α-SMA, which was consistently negative in NFs (n = 3) and positive in CAFs (n = 4). Representative figures of NFs and CAFs are shown in Fig. 1.

**Figure 2:**
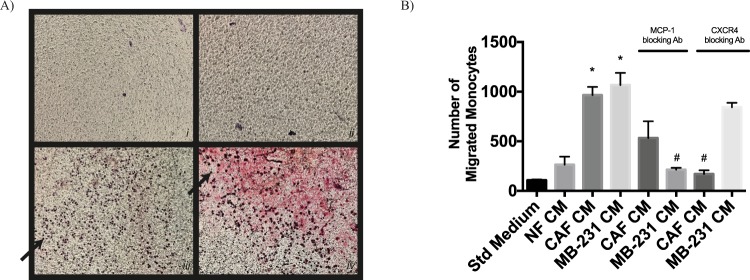
CAF and MDA-MB-231 cells effectively recruit monocytes. Monocytes isolated from healthy donors were serum starved and then were allowed to migrate for 5 h toward CMs from CAFs, NFs or MDA-MB-231 cells. (**A**) Representative microscopic views of each condition are shown (*i*: Standard Medium, *ii*: CM from NFs, *iii*: CM from CAFs, *iv*: CM from MDA-MB-231 Cells). (**B**) CAFs and MDA-MB-231 cells resulted in monocyte migration more than NFs did. The presence of CXCR4 (10 µg/ml) or MCP-1 (130 µg/ml) blocking antibodies diminished monocyte recruitment. ^*^p < 0.05 vs. standard medium, ^#^*p* < 0.05 vs. each corresponding control. Arrows demonstrate migrated/counted monocytes.

In order to evaluate the effects of CAFs and breast cancer cells on monocyte recruitment, we performed cell migration assays. Monocytes were isolated from peripheral blood of healthy humans. Conditioned mediums (CMs) collected from CAFs, NFs, and breast cancer cells were used in a Boyden migration assay, in which monocytes migrate toward CM in the bottom chamber. Our results demonstrated that CAFs as well as breast cancer cells recruited monocytes effectively (Fig. 2A,B).

### Recruitment of monocytes by CAFs might be mediated by MCP-1 as well as SDF-1 cytokines

In order to investigate the molecular mechanisms underlying monocyte recruitment by CAFs, we focused on MCP-1 and SDF-1 cytokines. Our results showed that inhibiting MCP-1 or SDF-1 activity through MCP-1 or CXCR4 (a chemokine receptor specific for SDF-1) blocking antibodies, respectively, reduced monocyte migration, significantly. Although both SDF-1 and MCP-1 are relevant for monocyte recruitment mediated by both CAF and breast cancer cells; SDF-1 seems to be more important for CAF-induced monocyte recruitment, while MCP-1 is more prominent for breast cancer cell mediated monocyte migration (Fig. 2B).

### CAFs induce a pro-tumoural phenotype, which resembles M2 macrophages like TAMs, in monocytes/macrophages in contrast to NFs

**Figure 3:**
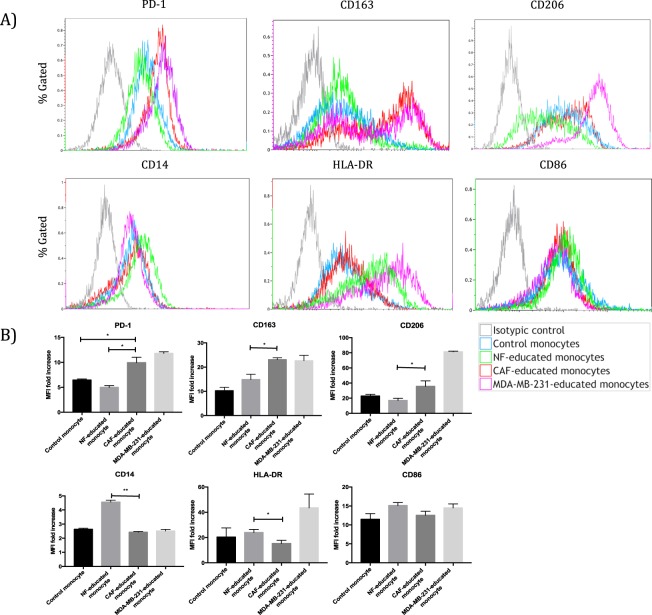
CAF and MDA-MB-231 cells induce monocytes to differentiate into a M2 phenotype similar to TAMs. CD14^+^ PBMCs isolated from healthy donors were cultured with CMs from NFs (NF-educated monocytes), CAFs (CAF-educated monocytes), MDA-MB-231 cells (MDA-MB-231-educated monocytes) or standard culture medium DMEM (control monocytes) for 7 days, then were analysed using flow cytometry. (**A**) The expression levels of PD-1, CD163, and CD206 were significantly higher in CAF-educated monocytes than in NF-educated monocytes. The expression levels of HLA-DR and CD14 were significantly lower in CAF-educated monocytes than in NF-educated monocytes. Representative histograms of each molecule are shown. (**B**) Data are presented as relative fold changes to control IgG in mean fluorescent intensity (MFI). *p < 0.05, **p < 0.01.

We established an *in vitro* model utilizing CD14^+^ cells isolated from healthy donors and CMs obtained from CAFs, NFs, and breast cancer cells in order to investigate the effects of the tumour stromal cells as well as the tumour cells on monocyte differentiation. Following 7 days of culture, the expressions of CD163 and CD206, which are mostly associated with M2 macrophages, were higher in CAF-educated cells than in NF-educated cells. In addition, the expression of programmed cell death protein 1 (PD-1) was higher in CAF-educated cells than in NF-educated cells. In fact, NF-educated cells’ expressions of PD-1, CD163 and CD206 were similar to control monocytes. On the other hand, the expression of CD14 was much higher in NF-educated cells than in CAF-educated cells. In addition, major histocompatibility complex (MHC) class II (HLA-DR) expression of CAF-educated cells is much lower than that of NF-educated cells as well as breast cancer cell-educated monocytes. The expressions of CD86 were comparable between the groups (Fig. 3).

### CAF-educated monocytes exhibit strong immunosuppression

**Figure 4:**
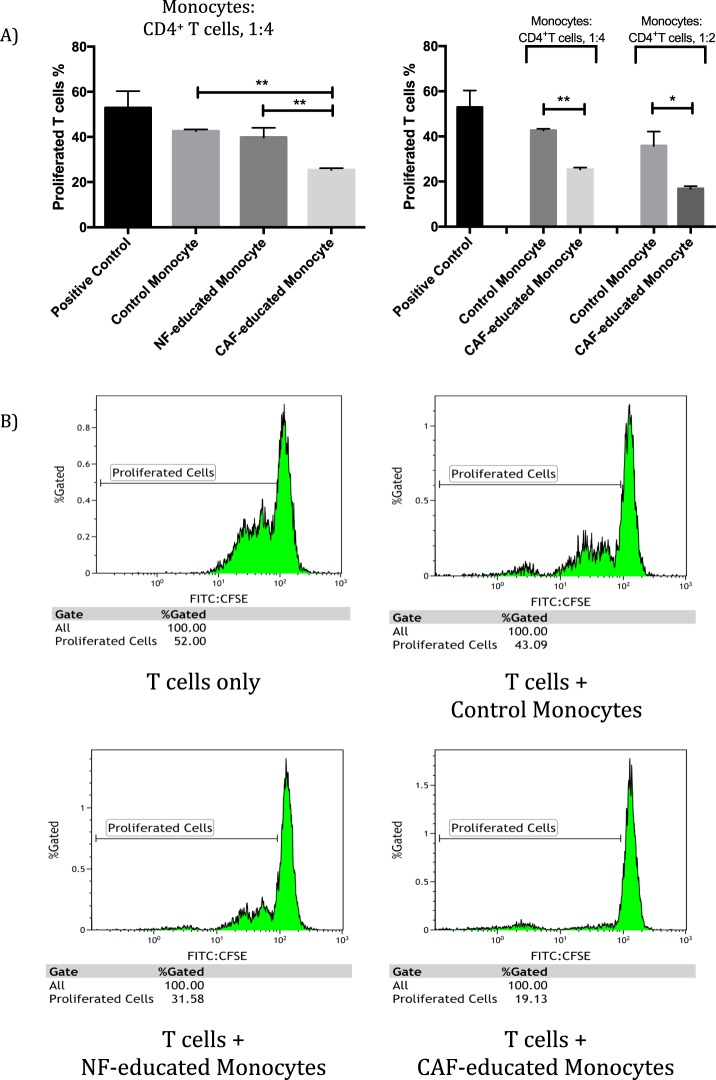
CAF-educated monocytes suppressed CD4^+^ T cell proliferation more than NF-educated monocytes did. CAF-educated monocytes, NF-educated monocytes, MDA-MB-231-educated monocytes, control monocytes were co-cultured with carboxyfluorescein succinimidyl ester (CFSE) labelled and autologous CD4^+^ T cells that were activated by CD3/CD28 magnetic beads for 96 hours. (**A**) The proliferation of T cells was suppressed by CAF-educated monocytes more strongly than control and NF-educated monocytes. Suppression of T-cell proliferation by CAF-educated monocytes is E:T dependent (normalized percentage of suppression of T-cell proliferation considering respective control monocytes are 40.1% and 53.3% for 1:4 and 1:2 E:Ts, respectively). (**B**) Representative histograms of the proliferation of T cells (monocytes:CD4^+^ T cells, 1:4). *p < 0.05, **p < 0.01.

We investigated whether CAF-educated monocytes could inhibit T-cell proliferation. For this purpose, human autologous CD4^+^ peripheral T lymphocytes stimulated with anti-CD3/CD28 magnetic beads were co-cultured with control monocytes, NF-, or CAF-educated monocytes. As shown in Fig. 4, CAF-educated monocytes markedly suppressed T-cell proliferation in effector-cell-to-target-cell-ratio (E:T) dependent manner; since a higher E:T seems to result in a more prominent immunosuppression (Fig. 4B).

### CAF-educated monocytes enhance the motility of breast cancer cells

**Figure 5:**
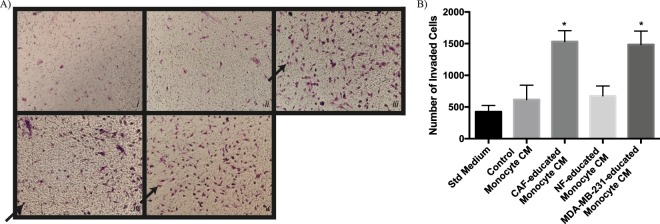
CAF-educated monocytes induce increased motility of MDA-MB-231 cells. Monocytes were cultured for 7 days with CMs from NF, CAF or MDA-MB-231 cells as well as in standard medium. All of the differentiated monocytes were then serum starved for 48 hours before obtaining the corresponding CMs. MDA-MB-231 cells were incubated with those CMs from differentiated monocytes for 24 hours. (**A**) Representative microscopic views of tumour cells that show invasion are shown (*i*: Standard Medium, *ii*: CM from Control Monocytes, *iii*: CM from CAF-educated Monocytes, *iv*: CM from NF-educated Monocytes, *v*: CM from MDA-MB-231-educated Monocytes). (**B**) Tumour cells that show invasion were counted and presented in bar graphs. *p < 0.05 vs. standard medium. Arrows demonstrate counted tumour cells that show invasion.

**Figure 6:**
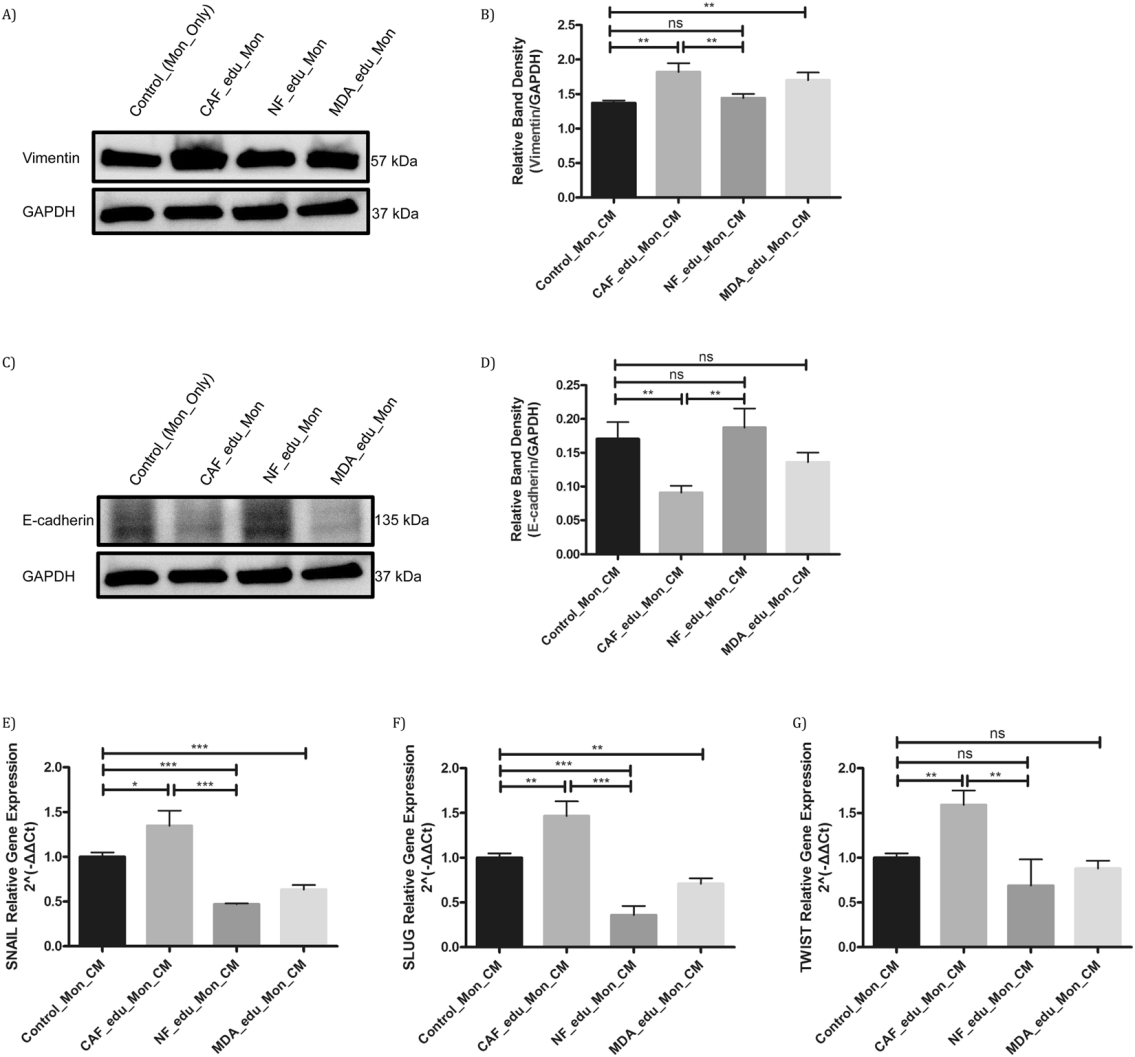
CAF-educated monocytes modify the expressions of EMT-related proteins and genes. Monocytes were cultured for 7 days with CMs from NF, CAF or MDA-MB-231 cells as well as in standard medium. All of the differentiated monocytes were then serum starved for 48 hours before obtaining the corresponding CMs. MDA-MB-231 cells were incubated with those CMs from differentiated monocytes for 24 hours. (**A**–**D**) The effects of NF-, CAF- or MDA-MB-231-educated monocyte CMs on the EMT of MDA-MB-231 breast cancer cells were analysed by Western blot analysis of vimentin and E-cadherin proteins. Densitometry shows relative protein expression normalized for GAPDH (n = 3). (**E**–**G**) The effects of NF-, CAF- or MDA-MB-231-educated monocyte CMs on the EMT of MDA-MB-231 breast cancer cells were analysed by quantitative real-time PCR of Snail, Slug and Twist gene expressions (n = 3). *p < 0.05. **p < 0.01. ***p < 0.001. ns: not significant.

In order to investigate the effects of CAF-educated monocytes on the motility of breast cancer cells, we treated MDA-MB-231 breast cancer cells with CMs from CAF-, NF-, or MDA-MB-231- educated monocytes. The invasion of breast cancer cells was enhanced by the effects of CAF-educated monocytes (similar to MDA-MB-231-educated monocytes), whereas the invasion of breast cancer cells was not significantly changed by the effects of NF-educated monocytes, compared to that of control monocytes (Fig. 5). In addition, we investigated the changes in expressions of EMT-related proteins of MDA-MB-231 breast cancer cells due to the effects of CAF-educated monocytes. For this reason, we treated MDA-MB-231 cells with CMs from CAF-, NF-, or MDA-MB-231- educated monocytes. Vimentin expression was found to be increased and E-cadherin expression was found to be decreased in breast cancer cells by the effects of CAF-educated monocytes. On the contrary, neither vimentin nor E-cadherin expression was significantly changed by the effects of NF-educated monocytes, compared to that of control monocytes (Fig. 6A–D). Real-time qPCR results showed that CAF-educated monocytes induce breast cancer cell expressions of Snail, Slug and Twist genes; which are EMT-related genes, both compared with control monocytes and with NF-educated monocytes (Fig. 6E–G).

### CAFs re-direct differentiated M1 macrophages to M2-like macrophages

**Figure 7:**
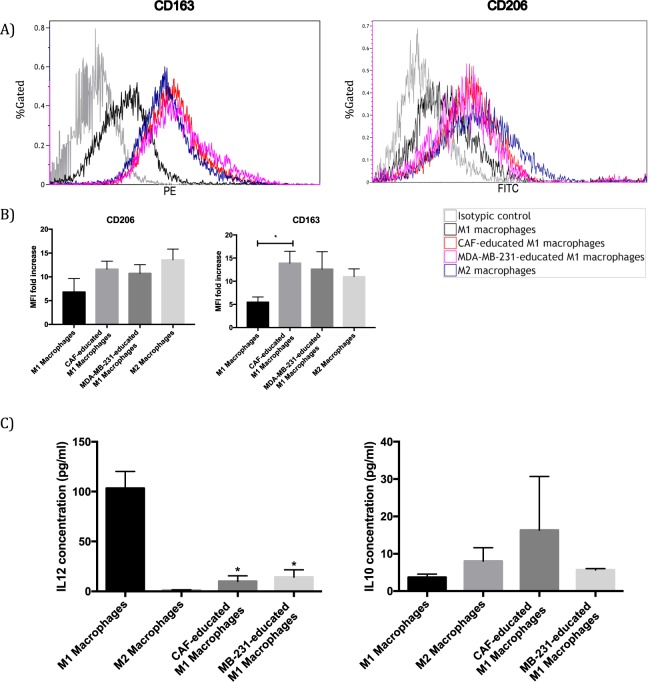
CAF and MDA-MB-231 cells induce M1 macrophages to differentiate into M2-like macrophages. (**A**) M1 macrophages were subsequently cultured with CMs from CAFs or MDA-MB-231 cells. After 48 hours, cells were analysed by flow cytometry. The expression levels of CD163 and CD206 were significantly higher in CAF- and MDA-MB-231-educated M1 macrophages than in M1 macrophages alone. Representative histograms of each molecule are shown. (**B**) Data are presented as relative fold changes to control IgG in mean fluorescent intensity (MFI). *p < 0.05. (**C**) M1 macrophages were cultured alone or with CMs from CAFs, or MDA-MB-231 cells for 48 hours and the levels of IL-12 or IL-10 were measured by ELISA. Levels of IL-12 was decreased in CAF- and MDA-MB-231-educated M1 macrophages compared with that in M1 macrophages alone. In contrast, levels of IL-10 increased in CAF- and MDA-MB-231-educated M1 macrophages compared with that in M1 macrophages alone. *p < 0.05.

We then set out to explore whether CAFs were able to induce the differentiation of M1 macrophages to M2 macrophages. For this reason, we cultured M1 macrophages with CMs of CAFs as well as breast cancer cells. After 2 days, the expression of CD163 significantly increased in M1 macrophages treated with CMs of either CAFs or MDA-MB-231 breast cancer cells; as a result, resembling the surface expression of M2 macrophages (Fig. 7). Even though CD206 expressions of CAF- or breast cancer cell-educated M1 macrophages slightly increased, such increases were not found to be statistically significant (Fig. 7B); whereas, CD206 expression of CAF-educated monocytes was shown to increase significantly (Fig. 3B). In addition, we determined IL-10 and IL-12 cytokine levels in culture supernatants. IL-12 cytokine levels decreased in the supernatants of both CAF- and breast cancer cell-educated M1 macrophages. On the other hand, IL-10 cytokine levels seem to have slightly increased in the supernatants of both CAF- and breast cancer cell-educated M1 macrophages, although this increase was not found to be statistically significant (Fig. 7C).

### High grade of CAFs significantly correlated with the number of TAMs in breast cancer tissue samples

We analysed the presence of macrophages positive for CD163 and CD206 as well as α-SMA positive fibroblasts in 50 human breast cancer samples using immunohistochemistry. We divided the sample group into high and low grade groups based on the median number of CD163^+^ macrophages (64; range 18−151), and CD206^+^ macrophages (15; range 2–50). We then analysed the associations of TAM grade with clinical and pathological features (Table 1). High level of infiltration of stromal CD163^+^ macrophages was associated with higher histologic grade, higher Ki-67 proliferation index, and higher tumour volume. High CAF grade was also associated with higher Ki-67 proliferation index, and higher tumour volume (Table 1).

**Table 1:** Correlation between the number of macrophages positive for CD163, and CD206 as well as α-SMA^+^ fibroblasts in breast tumour stroma with clinical and pathological features.

	No. of cases	CAF grade^‡^			CD163^+^ macrophages*			CD206^+^ macrophages*		
		Low (n = 27)	High (n = 23)	p value^†^	Low (n = 25)	High (n = 25)	p value^†^	Low (n = 28)	High (n = 22)	p value^†^
Age										
≤50	27	16	11	0.57	16	11	0.25	13	14	0.26
>50	23	11	12		9	14		15	8	
Grade										
2	8	6	2	0.26	7	1	**0.04**	3	5	0.27
3	42	21	21		18	24		25	17	
Ki-67 index^#^										
≤5%	4	4	0	**0.02**	4	0	**0.03**	3	1	0.60
>5%	25	8	17		9	16		13	12	
Lymph node involment										
Negative	26	13	13	0.58	13	13	1.00	16	10	0.56
Positive	24	14	10		12	12		12	12	
Tumor size										
≤2 cm	17	13	4	**0.03**	13	4	**0.01**	9	8	0.77
>2 cm	33	14	19		12	21		19	14	
Molecular subtypes										
ER/PR(+)	16	10	6	0.66	9	7	0.09	8	8	0.44
HER2(+)	11	6	5		8	3		8	3	
Triple negative	23	11	12		8	15		12	11	

*The median values of CD163^+^ or CD206^+^ macrophage numbers in stroma within the areas were used to divide the patients into high and low grade groups.

^‡^The grades of CAF area in stroma were used to divide the patients into two groups (low and high grade).

^#^Ki-67 index was evaluated in 29 tissue samples.

^†^Data were analysed by Fisher’s exact test and chi-squared test, and p < 0.05 was considered statistically significant.

**Figure 8:**
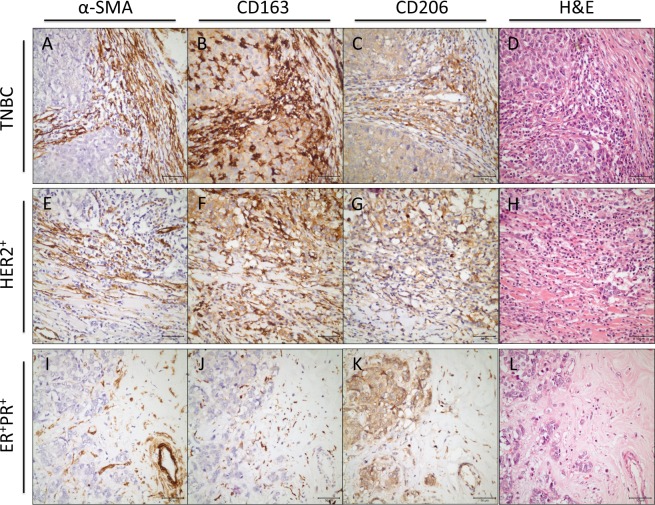
High grade of CAFs significantly correlated with the number of TAMs in breast cancer tissue samples. Immunohistochemical stainings for CD163, CD206, α-SMA, and Haematoxylin & Eosin (H&E) in breast cancer samples are shown. α-SMA (**A**), CD163 (**B**), CD206 (**C**), and H&E (**D**) were stained in a triple-negative breast cancer (TNBC) tissue sample. α-SMA (**E**), CD163 (**F**), CD206 (**G**), and H&E (**H**) were stained in a HER2^+^ breast cancer tissue sample. α-SMA (**I**), CD163 (**J**), CD206 (**K**), and H&E (**L**) were stained in an ER^+^PR^+^ breast cancer tissue sample (×400; scale bar, 50 µm). Sup. Figure 2 displays immunohistochemical stainings for laminin, CD34, and FAP. Three representative cases are shown.

As macrophages were observed to reside in the vicinity of α-SMA^+^ CAFs in the tissue samples (Fig. 8), we then investigated the association between the grade of TAMs and the grade of CAFs in the breast cancer tissues. Indeed, we found that high grade CAF tissues harboured higher numbers of CD163^+^ or CD206^+^ macrophages (Fig. 8A–D and Table 2). In addition, low CAF grade was associated with lower numbers of CD163^+^or CD206^+^ macrophages (Fig. 8I–L, Table 2).

**Table 2:** Correlation between macrophages positive for CD163, and CD206 with CAF grade in the stroma of breast cancer.

	No. of cases	CD163^+^ macrophages*			CD206^+^ macrophages*		
		Low (n = 25)	High (n = 25)	p value^†^	Low (n = 28)	High (n = 22)	p value^†^
CAF grade^‡^							
Low	27	23	4	<**0.001**	19	8	**0.04**
High	23	2	21		9	14	

*The median values of CD163^+^ or CD206^+^ macrophage numbers in stroma within the areas were used to divide the patients into high and low grade groups.

^‡^The grade of CAF area in stroma were used to divide the patients into two groups (low and high grade).

^†^Data were analysed by Fisher’s exact test and chi-squared test, and p < 0.05 was considered statistically significant.

### CAF-educated monocytes increased the proliferation of breast cancer cells

**Figure 9:**
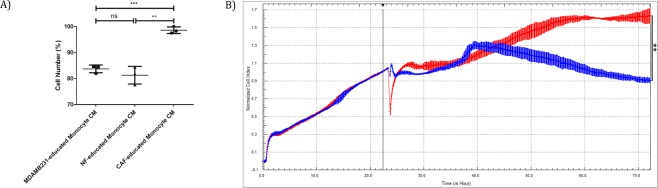
CAF-educated monocytes induce increased proliferation of MDA-MB-231 cells. Monocytes were cultured for 7 days with CMs from NF or CAF cells. All of the differentiated monocytes were then serum starved for 48 hours before obtaining the corresponding CMs. (**A**) Proliferation of MDA-MB-231 breast cancer cells were evaluated with MTT assay after 48 hours of treatment with corresponding CMs and normalized cell numbers are shown (mean ± SD, n = 3). (**B**) MDA-MB-231 cells were seeded in an ACEA E-plate. After a day of incubation, they were incubated with those CMs from differentiated monocytes for an additional 48 hours. Cell index was measured by electrical impedance (n = 3). Normalized cell index (22:26:25, mean ± SD) as a measure for cell proliferation of MDA-MB-231 cells that were treated with NF- (blue) or CAF-educated (red) monocyte CMs is shown. Vertical line demonstrates the time of normalization. **p < 0.01. ***p < 0.001. ns: not significant.

In order to investigate the effects of CAF-educated monocytes on breast cancer cell proliferation *in vitro*, we treated MDA-MB-231 breast cancer cells with CMs from CAF- or NF- educated monocytes. CAF-educated monocytes increased the proliferation of breast cancer cells. On the other hand, the proliferation of breast cancer cells was not significantly changed by the effects of NF-educated monocytes, compared to that of MDA-MB-231- educated monocytes (Fig. 9A). Similar findings concerning the effects of NF- and CAF- educated monocytes on breast cancer cell proliferation were observed in real-time and label-free analysis of MDA-MB-231 cells with the xCELLigence system (Fig. 9B).

## Discussion

It is now clear that there is a continual crosstalk not only between tumour cells and immune cells but also between stromal cells such as CAFs and immune cells. This crosstalk has an important role on development and progression of tumours. CAFs have recently been implicated in regulation of immune cell recruitment and function. In this study, we aimed to determine the role of cancer associated fibroblasts, compared with normal fibroblasts and breast cancer cells, on monocyte recruitment and polarization in breast cancer.

The perspective concerning adult tissue macrophages being solely derived from bone marrow precursor cells have been recently abandoned. In fact, it has been shown that most tissue resident macrophages originated from yolk sac precursor cells^18,19^. On the other hand, macrophages found in pathogen associated inflammation mostly originate from bone marrow monocytes^20^. It has recently been show that most TAM sub-populations emerge from the Ly6C^+^ population of circulating mouse monocytes in grafted tumours^21^, primary mouse mammary tumours^22^ and in lung metastases^23^. Therefore, investigating the factors associated with monocyte recruitment to tumour tissues has utmost importance. Previous studies have shown that CAFs secrete large amounts of SDF-1. We found that CAF derived SDF-1 was crucial for monocyte recruitment, which underlines the key importance of the SDF-1/CXCR4 axis for progression of several cancers^24^. Previous studies have also shown that MCP-1 was able to induce infiltration of blood monocytes into CAF spheroids and to recruit monocytes into mammary tumours^25^. Even though CAF-induced monocyte migration decreased with MCP-1 inhibition in our study, this decrease was not as prominent as that seen with SDF-1 inhibition. This finding demonstrated that SDF-1 is more significant in CAF-induced monocyte chemotaxis than MCP-1.

Programmed cell death protein 1 (PD-1) is an immune checkpoint receptor that is upregulated on activated T cells for the induction of immune tolerance^26,27^. Monoclonal antibodies targeting PD-1/PD-L1 pathway have been shown to be effective against several types of cancer^28^. In a very recent study, Gordon *et al*. have shown that PD-1 is also expressed by both mouse and human TAMs in addition to activated T cells^29^. They also characterized PD-1^+^ TAMs and showed that almost all PD-1^+^ TAMs express an M2-like surface profile^29^. In light of this important study, we investigated whether CAFs might have a role in inducing such immunosuppressive PD-1^+^ TAMs. The role of CAFs in the induction of PD-1 expression on TAMs have not been investigated before. We found that CAF-educated monocytes demonstrated high PD-1 expression as well as CD163 and CD206 expressions. Our findings demonstrate for the first time that CAFs are able to induce a PD-1^+^ TAM phenotype by themselves, even without the presence of tumour cells. In addition, our findings prove that CAFs are as potent as tumour cells in terms of inducing such PD-1 expressions (Fig. 3). Furthermore, we also demonstrated that PD-1^+^ TAMs are relevant for breast cancer in addition to colorectal cancer^29^. In line with these findings, we suggest that CAFs may recruit monocytes and induce these recruited monocytes to differentiate into PD-1 expressing M2-like macrophages, instead of M1 macrophages. Therefore, PD-1 axis might be crucial in CAF induced immune suppression *in vivo* in several types of cancer including breast and colorectal tumours.

In the current study, we also showed that CAF-educated monocytes exhibited increased expression of CD206 and CD163. Immune functions of CD206 (C-Type Mannose Receptor 1) has not yet been fully understood. It has been shown that the lack of CD206 results in the upregulation of pro-inflammatory cytokine production during endotoxemic lung inflammation in mice^30^ and increased serum levels of inflammatory proteins, suggesting that it may have a role in the resolution of inflammation by decreasing inflammatory molecules in the blood^31^. CAF mediated induction of higher expression of CD206, which has anti-inflammatory effects, on monocytes may also demonstrate another indirect mechanism of immune suppression by CAFs. CD163 is a haptoglobin-hemoglobin scavenger receptor and it correlates with known prognostic factors (e.g. poor differentiation (Grade 3), ER negativity and ductal type which are associated with bad prognosis)^32^. As one would expect, CAFs induced a very high CD163 expression on monocytes compared with NFs (Fig. 3). Interestingly, we found that breast cancer cells induced high CD163 and CD206 expressions on monocytes as well as MHC class II (HLA-DR) expression; whereas CAFs did not induce HLA-DR expression on monocytes. HLA-DR is well known to be associated with M1 macrophages. The induction of HLA-DR expression on monocytes by breast cancer cells may seem controversial; however, the study by Edin *et al*. has shown that colorectal cancer cells induce TAMs of a “mixed” M1/M2 phenotype, which in turn could contribute to a “good inflammatory response”^33^. Moreover, Helm *et al*. have demonstrated that HLA-DR and CD163 double positive cells were detectable by immunohistochemical stainings in pancreatic ductal adenocarcinoma tissues^34^. We propose that CAF-mediated down regulation of MHC class II expression on monocytes may demonstrate a mechanism of immune escape via decreased antigen presentation, in addition to confirming the notion that CAFs are not implicated in any types of “good inflammatory response”. On the other hand, it should also be noted that NF-educated monocytes expressed both CD14 and HLA-DR, which are associated with M1 macrophages^3,4^, more than CAF-educated monocytes. This finding shows that activated tumour stroma may have explicit functions in terms of macrophage polarization. In line with our findings, Zhang *et al*. demonstrated that the number of CD68^+^CD163^+^ macrophages was higher in breast cancer tissues than in normal breast tissues^35^.

Several studies showed that TAMs promote breast cancer invasion and metastasis by releasing a variety of cytokines^36–38^. In the current study, we demonstrated that CAF-educated monocytes increased breast cancer cell invasion as well as the expressions of EMT-related genes and vimentin protein significantly, while decreasing E-cadherin protein expression; in contrast to NF-educated monocytes. Our *in vitro* migration assay findings and gene/protein expression results display that CAF-educated monocytes enhance the motility of breast cancer cells. In a very recent study by Linde *et al*., CD206^+^ macrophages were shown to downregulate E-cadherin junctions in breast cancer cells^39^, which may facilitate EMT. Thus, our findings support the notion that CAFs promote EMT, invasion and metastasis^40^; via inducing M2 macrophages. We then investigated whether CAF-educated monocytes have immunosuppressive features. CAF-educated monocytes significantly suppressed T cell proliferation in contrast to control or NF-educated monocytes. CAFs were known to directly inhibit proliferation of T lymphocytes^16,41^. In the current study, we showed that CAFs also exert immunosuppressive effects, indirectly, by differentiating the monocytes into M2-like TAMs.

Activation status of M1/M2 macrophages in tumour tissues is transient, which gives them a functional plasticity^42^. Our results suggest that CAFs may re-direct M1 macrophages to M2-like macrophages in the tumour tissue. The differentiation of macrophages from anti-tumoural M1-like to pro-tumoural M2-like macrophages is a crucial event in the establishment of the tumour microenvironment. Thus, investigating the factors that drive the trans-differentiation of pro-inflammatory M1 macrophages to anti-inflammatory M2 macrophages in tumour tissues has great importance in terms of targeted therapies^12^. We observed that CAF-educated M1 macrophages showed increased secretion of IL-10 and decreased secretion of IL-12 in addition to phenotypic changes (i.e. increased expression of M2 markers). Even though our results demonstrated that CAF-educated M1 macrophages seem to display increased expressions of both CD163 and CD206, the increase in the expression of CD206 did not reach statistical significance (Fig. 7B). Given our results showing that CAF-educated monocytes show significantly increased expressions of both CD163 and CD206 (Fig. 3B), CD206 expression change of CAF-educated M1 macrophages seems interesting. In fact, our studies with human breast cancer tissue samples also demonstrated that higher number of CAFs is correlated with higher numbers of not only CD163^+^ but also CD206^+^ macrophages (Table 2). CD206 was reported to be more associated with M2a-like macrophages, while CD163 is prominent on the surface of M2c-like macrophages^43,44^. In addition, Mantovani *et al*. reported that M2a macrophages are associated with T_H_2 type immune responses and elimination of parasites, while M2c macrophages are more implicated in immunoregulation and tissue remodelling^45^. Therefore, CAFs may be inclined to polarize the M1 macrophages more into the M2c phenotype that is associated with immune suppression than M2a. Such a predilection seems reasonable considering tumours are known as wounds that do not heal and thus require a chronic wound healing/tissue remodelling process instead of an allergic response or killing of parasites. This might explain our results about CAF-educated M1 macrophages and our findings suggest that CAFs may be influencing M1/M2 trans-differentiation. It has been shown that IL-4, which is secreted from tumour cells and T lymphocytes^46^, as well as colony stimulating factor 1 (CSF-1) and granulocyte-macrophage colony-stimulating factor (GM-CSF), which are secreted from tumour cells^47,48^, take part in pro-tumoural differentiation of macrophages. In line with these studies, we suggest that CAFs may also prove to be important players in this re-polarization process.

Since the number of α-SMA^+^ fibroblasts was found to be significantly higher in the tumour stroma compared with that in benign breast tissue^49^ and the number of TAMs was higher in tumour stroma than in tumour nest^50^, we analysed the tumour stroma to evaluate both of these cells rather than the tumour nest. We found that the number of CD163^+^ TAMs significantly correlated with high tumour grade, high Ki-67 proliferation index and high tumour size. In line with such findings, CAF-educated monocytes were found to increase breast cancer cell proliferation *in vitro* (Fig. 9). The infiltration of CD163^+^ TAMs seemed to be the highest in triple negative, and the lowest in ER^+^PR^+^ breast cancer samples (Fig. 8), although such findings were not statistically significant.

The copresence of CAFs and TAMs inside a tumour was considered to be a potential prognostic feature in colorectal cancer patients^51^. In the current study, we showed that there exists a significant correlation between the number of TAMs and the CAF grade in breast cancer. It was recently reported that CAF-derived Chitinase 3-like 1, which is implicated in inflammatory disorders, contributed to tumour growth in breast cancer and this contribution is accompanied by a high infiltration of M2-polarized macrophages and T_H_2 type immune responses^52^. Therefore, our finding of increased infiltration of breast tumour tissues with TAMs might be a direct result of the presence of high number of CAFs in those tumours; since we also demonstrated that CAFs recruit monocytes and induce M2 type polarization.

In this study, we examined the relationship between cancer-associated fibroblasts (CAFs) and monocytes/macrophages in breast cancer using human *ex vivo* cellular systems and tissues samples obtained from breast cancer patients as well as *in vitro* cell lines. Even though our studies were not performed using *in vivo* systems, our results demonstrate that CAFs promote monocyte migration and enhance their differentiation to M2-like macrophages; based on phenotype, cytokine and functional features. In addition, our studies with human breast cancer patient tissue microarrays (TMAs) showed some translational promise. Future *in vivo* experiments will shed further light on such new mechanistic insights and provide broader implications, as such systems would simulate all the elements of the tumour microenvironment, as well as demonstrating the effects of cell-to-cell contact in addition to the cross-talk mediated via soluble factors demonstrated in our study. Accordingly, *in vivo* experiments in severe combined immunodeficiency (SCID) mice can be used to prove that human monocytes co-injected s.c. with tumour cells acquire a stronger protumoural effect if preconditioned with CAFs in contrast to NFs.

## Materials and Methods

### Isolation of fibroblasts from normal and cancerous breast tissue

Normal breast tissues were obtained from 3 patients undergoing reduction mammoplasty and breast cancer tissues were obtained from 4 patients undergoing therapeutic mastectomy. All fibroblasts were isolated using a previously described protocol^41,53^ that utilizes Collagenase I (1 mg/mL) and Hyaluronidase (125 U/mL) enzymes followed by differential sedimentation and plating. Enzymatically digested tissues were then cultured in high serum media conditions which select for fibroblast growth (Dulbecco’s Modified Eagle’s medium [DMEM] supplemented with 10% foetal bovine serum at 37 C, 5% CO_2_). All human breast tissues for all of the experiments were obtained in accordance with the laws and institutional guidelines, as approved by the institutional review board of Hacettepe University (Approval numbers: GO 16/64 - 20 and GO 17/380 - 31). Informed consent was obtained from all participants.

### Characterization of fibroblasts with immunocytochemistry

Characterizations of fibroblasts were performed by immunocytochemistry stainings with vimentin, pan-cytokeratin and α-SMA according to the manufacturer’s instructions. Cells (7 × 10^4^ cells/300 µL in each well) were cultured in eight well chamber slides and immunocytochemical / morphological analyses were performed when the cells reached a surface confluency of >80%. A biotin/streptavidin/horseradish peroxidase detection system was utilized and binding of the antibody was demonstrated with diaminobenzidine (DAB) substrate. The images were captured by Olympus BX50 microscope.

### Preparation of conditioned medium (CM)

CMs were obtained from CAFs, NFs and MDA-MB-231 breast cancer cells. When the aforementioned cells reached to a confluency of >80%, they were serum starved. After 48 h, CMs were collected from cell supernatants and clarified by centrifugation.

### Isolation and culture of peripheral CD14^+^ monocytes

PBMCs were isolated from healthy volunteers’ peripheral blood using density gradient separation with Histopaque-1077. Separation of CD14^+^ monocytes from PBMCs was accomplished by using a magnetic bead based positive selection protocol with a purity of >95%. CD14^+^ monocytes were cultured alone as control monocytes or with CMs from CAFs, NFs or MDA-MB-231 breast cancer cells (CM:RPMI, 1:1) for 7-days.

### Phenotypical analyses with flow cytometry

CD14^+^ PBMCs isolated from healthy donors were cultured with CMs from NFs, CAFs, MDA-MB-231 breast cancer cells as well as in standard medium for 7 days, then analysed using flow cytometry. M1 macrophages were cultured with CMs from CAFs or MDA-MB-231 cells for 48 hours, then analysed by flow cytometry.

Cultured monocytes or macrophages were detached using Accutase cell detachment solution, according to the manufacturer’s instructions. Cells were stained with antibodies against CD206, CD163, HLA-DR, CD14, and PD-1. All antibodies were conjugated to Phycoerythrin (PE) or Fluorescein isothiocyanate (FITC). Respective isotype-matched antibody controls were used as negative controls.

### Isolation of peripheral blood CD4^+^ T-cells

PBMCs were isolated from healthy volunteers’ peripheral blood using density gradient separation with Histopaque-1077. Separation of CD4^+^ T-cells from PBMCs was accomplished by using a magnetic bead based negative selection protocol with a purity of >95%. CD4^+^ T-cells were cultured in Roswell Park Memorial Institute (RPMI) 1640 medium supplemented with 10% foetal calf serum, 2.1 mM L-glutamine, 100 U/mL penicillin and 100 mg/mL streptomycin at 37 °C, 5% CO_2_.

### CFSE proliferation assays

In order to assess the effects of CAF-, NF- and MDA-MB-231-educated monocytes on CD4^+^ T lymphocytes, we performed functional analyses with flow cytometry. Carboxyfluorescein succinimidyl ester (CFSE) labelled CD4^+^ T-cells were activated by CD3/CD28 magnetic beads (except the negative control group). Activated T-cells were cultured with either control monocytes or with monocytes that had been cultured with CMs from CAFs, NFs or MDA-MB-231 cells for 7 days. After 96 hours, proliferation of CFSE labelled CD4^+^ T cells was evaluated by flow cytometry.

### Evaluation of cell migration

In order to evaluate the effects of CAFs, NFs, or MDA-MB-231 cells on monocyte recruitment, we performed *in vitro* migration assays. Monocyte cells were seeded into the upper compartment in serum-free medium (5 × 10^4^ cells in 200 µl). The 5 µm Transwell chambers were placed into 24-well culture wells containing CMs from CAFs, NFs, or MDA-MB-231 cells. After 5 h incubation, non-migrated cells were removed by cotton swab. Migrated cells were fixed by methanol and stained with Crystal violet. Migrated cells were counted in five different fields of polycarbonate filter under a microscope (magnification x100).

### Evaluation of EMT-related protein expressions by Western Blot

MDA-MB-231 breast cancer cells were cultured for 24 hours prior to CM treatments. The cells were then treated with CMs from NF-, CAF-, MDA-MB-231- educated monocytes or from control monocytes for 48 hours. At the end of this period, cells were lysed in RIPA (Thermo Fisher Scientific, Waltham, MA, USA) lysis buffer supplemented with protease and phosphatase inhibitor cocktail (Thermo Fisher Scientific, Waltham, MA, USA). E-cadherin (primary antibody: Cell Signaling Technology, Danvers, MA, USA; #3195) and vimentin (primary antibody: Cell Signaling Technology, Danvers, MA, USA; 5741) protein expressions in samples were examined by the Western blot method. GAPDH (primay antibody: Cell Signaling Technology, Danvers, MA, USA, #5174) was used as the Western blot loading control. The membranes were visualized by chemiluminescence (ECL) and densitometry analyses of the protein bands were performed.

### Evaluation of EMT-related gene expressions by quantitative real-time PCR

MDA-MB-231 breast cancer cells were cultured for 24 hours prior to CM treatments. The cells were then treated with CMs from NF-, CAF-, MDA-MB-231- educated monocytes or from control monocytes for 48 hours. At the end of this period, RNA isolation from the cells was performed according to the Zymo Quick-RNA Miniprep Kit protocol. Complementary DNA was synthesized with Thermo Scientific RevertAid First Strand cDNA Kit according to the manufacturer’s instructions. Real-time qPCR was performed using LightCycler FastStart DNA Master SYBR Green (Roche, Basel, Switzerland). The qPCR data were analysed using the Livak model (2^−ΔΔCt^). Snail (Snail_reverse 5′-CTGCTGGAAGGTAAACTCTGGA-3′, Snail_forward 5′-CGAGTGGTTCTTCTGCGCTA-3′), Slug (Slug_reverse 5′-TTCTCCCCCGTGTGAGTTCTAA-3′, Slug_forward 5′-CACTGCGATGCCCAGTCTA-3′), and Twist (Twist_reverse 5′-CCCACGCCCTGTTTCTTTGA-3′, Twist_forward 5′-GCCGGAGACCTAGATGTCATT-3′) gene expressions were evaluated. β -actin was used as the reference gene.

### Evaluation of cell invasion

MDA-MB-231 breast cancer cell invasion was evaluated by using Transwell inserts *in vitro*. Monocytes were differentiated for 7 days using CMs from NF, CAF or MDA-MB-231 cells as well as in standard medium. All the differentiated monocytes were then serum starved for 48 h and corresponding supernatants were then collected. MDA-MB-231 cells in serum-free medium were seeded into upper compartments of Transwell inserts which were pre-coated with Matrigel (5 × 10^4^ cells in 200 µl). The lower compartment contained the aforementioned supernatants, which were obtained from monocytes. After 24 hours, non-migrated cells were removed by cotton swab. Migrated cells were fixed by methanol and stained with Crystal violet. Migrated cells were counted in five different fields of polycarbonate filter under a microscope (magnification x200).

### Induction of M1/M2 polarization of macrophages

CD14^+^ monocytes were cultured in medium containing macrophage colony-stimulating factor (M-CSF) (50 ng/ml) for 7 days to obtain M0 macrophages. In order to acquire M1 macrophages, M0 macrophages were further cultured in medium containing interferon gamma (IFNγ) (100 ng/ml) and lipopolysaccharides (LPS) (10 ng/ml) for 2 more days. On the other hand, M0 macrophages were further cultured in medium containing IL-4 (20 ng/ml) for 2 more days in order to obtain M2 macrophages.

### Assessment of soluble factors by ELISA

In order to evaluate the levels of IL-12 and IL-10 cytokines in the culture supernatants of M1 and M2 macrophages, we performed enzyme-linked immunosorbent assays (ELISA). We cultured M1 or M2 macrophages alone (as negative control) or with CMs from CAFs, or MDA-MB-231 breast cancer cells for 2 days. After this period, the cells were further cultured for additional 2 days in serum-free medium. Culture supernatants were stored at −80 °C until the assays were performed.

### Measurement of cell proliferation by MTT assay

MDA-MB-231 breast cancer cell proliferation was evaluated via MTT (3-(4, 5-dimethylthiazolyl-2)-2, 5-diphenyltetrazolium bromide) assay. MDA-MB-231 cells were seeded in 96-well plates. After about a day of incubation, CMs obtained from CAF- or NF-educated-monocytes were added to the wells and MDA-MB-231 cell proliferations were analysed after an additional 48 hours (72 hours in total) by adding 25 μl MTT reagent (final concentration: 1 mg/ml) in each well. 4 hours after adding the MTT reagent, the precipitate was solubilized by 80 μl detergent reagent. The plates were then left overnight in the dark and the absorbances were recorded at 570 nm.

### Real-time and label-free analysis of MDA-MB-231 cells with the xCELLigence system

In order to assess the real-time effects of CAF- and NF-educated monocytes on MDA-MB-231 breast cancer cell proliferation, we performed functional analyses with the RTCA xCELLigence System (ACEA, San Diego, CA, USA). This system utilizes gold biosensor microelectrodes to measure electrical impedance. The background impedance of the microelectrodes were first measured with cell culture medium. Then, MDA-MB-231 breast cancer cells were added to each well of the E-plate. After about a day of incubation, CMs obtained from CAF- or NF-educated-monocytes were added to the wells and the cell indices were measured by electrical impedance over an additional period of 48 hours (72 hours in total).

### Tissue samples and immunohistochemical stainings

A total of 50 human invasive breast cancer tissue samples were investigated. Patients who had received preoperative/adjuvant chemotherapy or radiotherapy were excluded. Clinical and pathological variables including age, tumour grade and size, lymph node involvement, Ki-67 staining as well as ER, PR and HER2 status were evaluated.

Formalin-fixed paraffin-embedded tumour samples were used for tissue microarrays (TMAs). Blocks were made utilizing a 4.0 mm tissue cylinder through a histologically representative area of each donor tumour block. From each donor block, 1–2 cores were cut and 8 TMA blocks were prepared. Immunohistochemical stainings for CD163 (Biocare, Clone 10D6, 10 min EDTA, 1:100), CD206 (Abcam, ab64693, 20 min EDTA, 1:200) and α-SMA (Neomarkers, Clone 1A4, 1:1000) as well as Haematoxylin and Eosin (H&E) were performed on the 4-μm thick sections taken from the tissue microarrays using Leica Bond Max III Autostainer, following manufacturer’s instructions. Concentrations and antigen retrieval methods were optimized for each antibody using appropriate positive and negative controls.

### TAM and CAF grade evaluations

All tissue microarray slides stained with anti-CD163 or anti-CD206 were analysed to identify areas with the highest levels of TAM infiltration. For each case, three hot spots in the tumour stroma (40x objective) were selected for counting TAMs. The median values of CD163^+^ or CD206^+^ macrophage numbers in stroma within the areas were used to categorize the patients into high and low grade groups. The intensity of CAFs (α-SMA positive spindle-shaped fibroblasts) was classified into 2 grades, using a modification of a method previously described^54^: negative to scanty (low grade) or abundant (high grade) in a blinded analysis performed by 2 of the authors (K.K., and B.G.Y.).

### Statistical analyses

The Mann-Whitney U test, Student’s t-test, chi-squared test, and Fisher’s exact test were used to examine differences in continuous and categorical variables. A 5% type-I error level was used to infer statistical significance. All statistical analyses were carried out using IBM SPSS Statistics for Windows Software version 23.

## Conclusions

Our results propose that CAFs recruit monocytes and induce TAMs which might be suppressing the immune system via the PD-1 axis. We herein demonstrated that CAFs play pivotal roles in sculpturing the tumour microenvironment in breast cancer, and therapeutic strategies to reverse the CAF-mediated immunosuppressive microenvironment should be taken into consideration in order to increase the effectiveness of conventional therapies as well as immunotherapies against breast cancer.

## Supplementary information

Supplementary Information

## Data Availability

All data generated or analysed during this study are included in this published article.
