# Supplementary material for: Cancer associated fibroblasts sculpt tumour microenvironment by recruiting monocytes and inducing immunosuppressive PD-1+ TAMs
Source: Sci Rep. 2019 Feb 28;9:3172. doi: 10.1038/s41598-019-39553-z (PMC6395633; doi:10.1038/s41598-019-39553-z)
Supplement: Supplementary file 1 — Supplementary Information [file 41598_2019_39553_MOESM1_ESM.pdf]

Supplementary Information

for

Cancer associated fibroblasts sculpt tumour microenvironment by recruiting monocytes and inducing immunosuppressive PD-1^{+} TAMs

Betul Gok Yavuz[1], Gurcan Gunaydin[1], M. Emre Gedik[1], Kemal Kosemehmetoglu[2], Derya Karakoc[3], Figen Ozgur[4] and Dicle Guc[1]

[1]*Department of Basic Oncology, Hacettepe University Cancer Institute, Sihhiye, 06100, Ankara, Turkey*

[2]*Department of Pathology, Hacettepe University School of Medicine, Sihhiye, 06100, Ankara, Turkey*

[3]*Department of General Surgery, Hacettepe University School of Medicine, Sihhiye, 06100, Ankara, Turkey*

[4]*Department of Plastic Reconstructive and Aesthetic Surgery, Hacettepe University School of Medicine, Sihhiye, 06100, Ankara, Turkey*

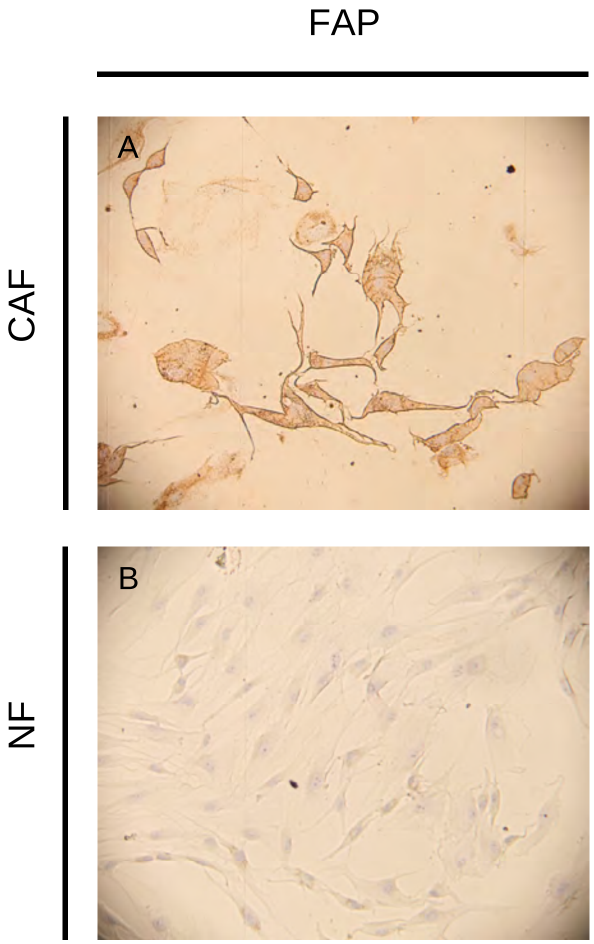

Sup. Figure 1

Sup. Figure 1. Immunocytochemical characteristics of NF and CAF cells. CAFs and NFs were cultured in 8-well chamber slides. CAFs (A) and NFs (B) were stained with anti-fibroblast activation protein (FAP) antibody. One representative figure for each cell type is shown (×100).

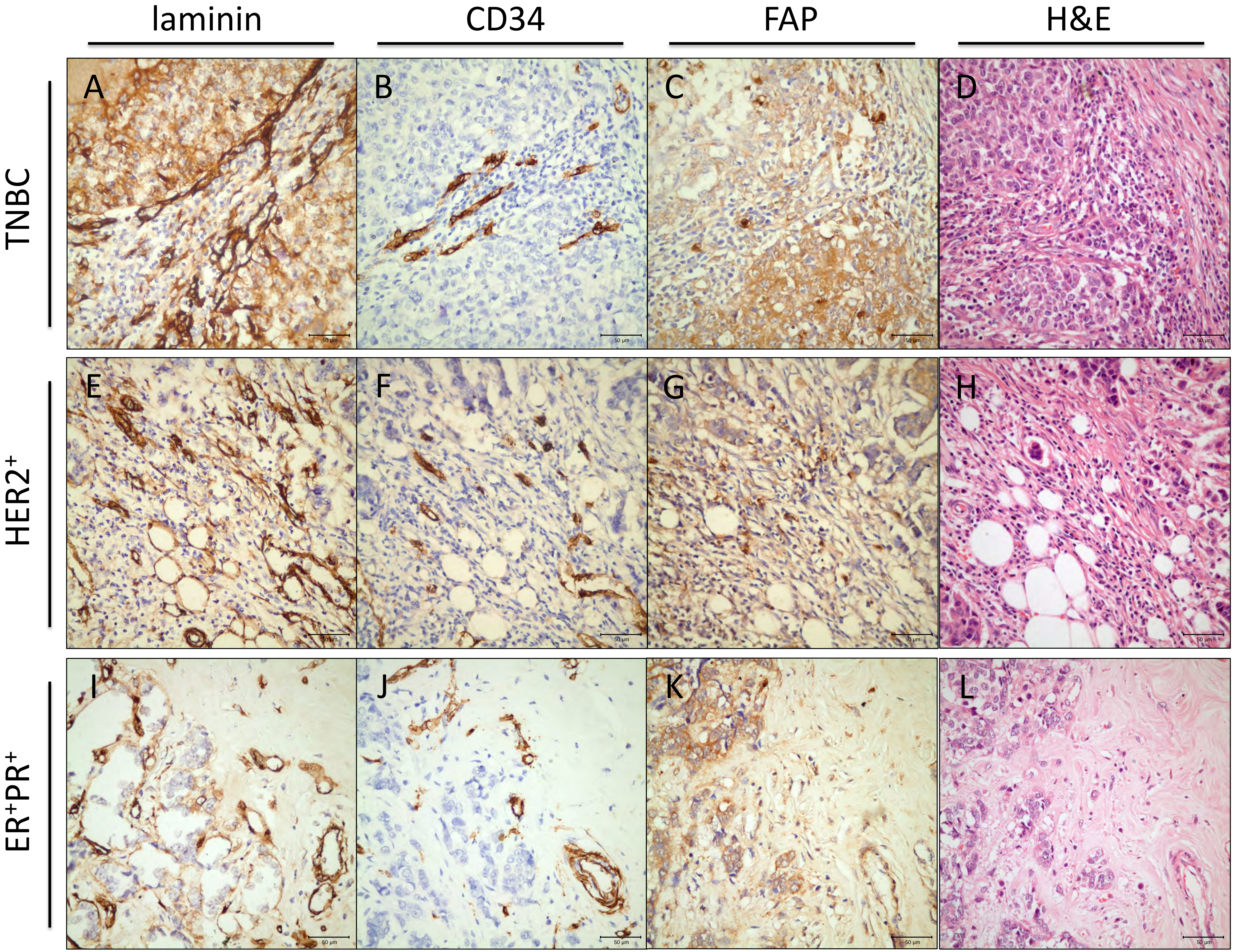

Sup. Figure 2

Sup. Figure 2. Immunohistochemical characteristics of breast cancer tissue samples. Immunohistochemical stainings for laminin (Novocastra, 1:50), CD34 (Leica Biosystems, 1:400), FAP (St John's Laboratory, 1:200), and Haematoxylin & Eosin (H&E) in breast cancer samples are shown. Laminin (A), CD34 (B), FAP (C), and H&E (D) were stained in a triple-negative breast cancer (TNBC) tissue sample. Laminin (E), CD34 (F), FAP (G), and H&E (H) were stained in a $HER2^+$ breast cancer tissue sample. Laminin (I), CD34 (J), FAP (K), and H&E (L) were stained in an ER^+PR^+ breast cancer tissue sample (×400; scale bar, 50 μm). Three representative cases are shown.

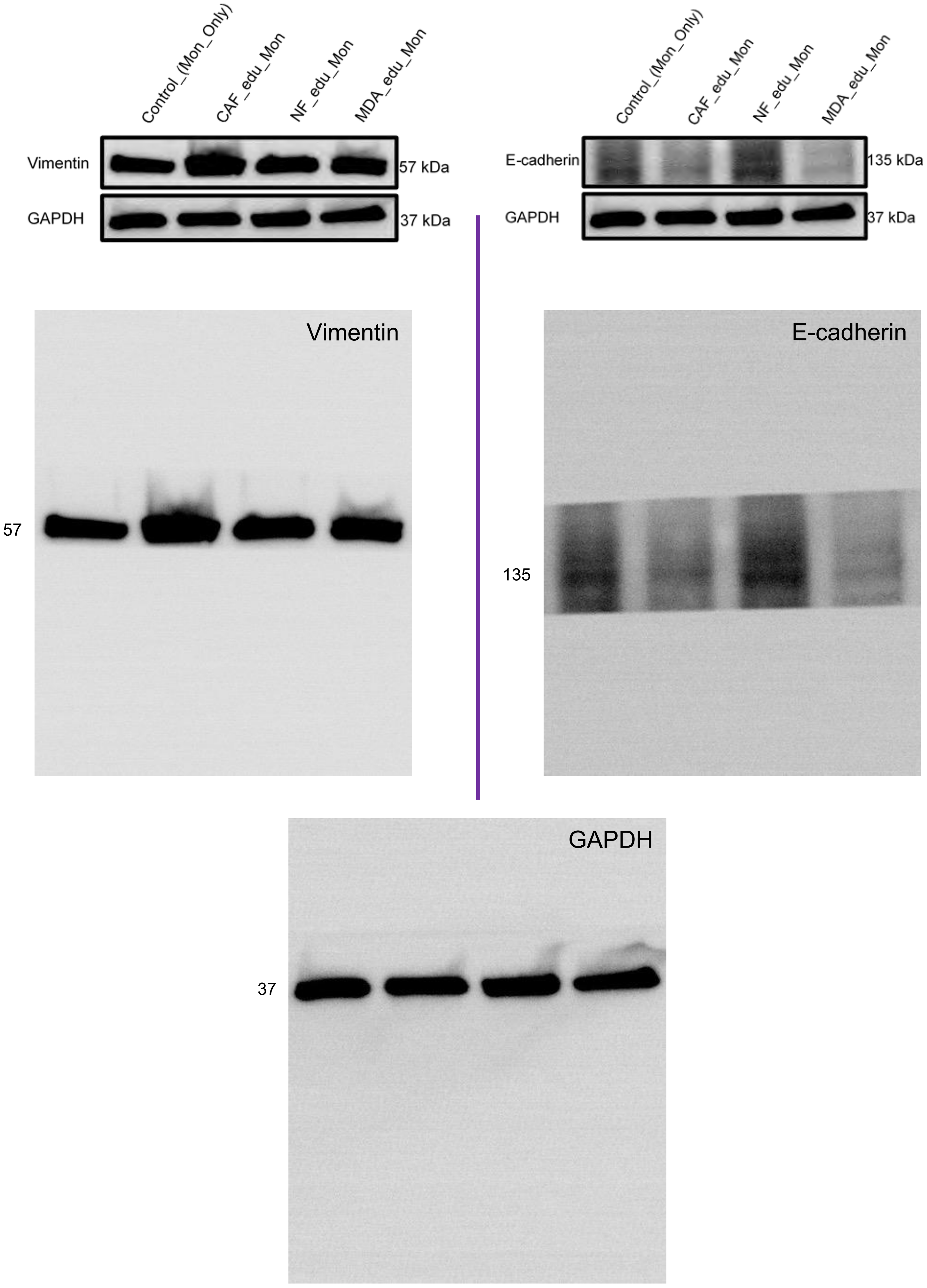
Control_(Mon_Only)
CAF_edu_Mon
NF_edu_Mon
MDA_edu_Mon
Vimentin
57 kDa
GAPDH
37 kDa
Control_(Mon_Only)
CAF_edu_Mon
NF_edu_Mon
MDA_edu_Mon
E-cadherin
135 kDa
GAPDH
37 kDa
Vimentin
57
E-cadherin
135
GAPDH
37

Sup. Figure 3

Sup. Figure 3. Original immunoblots for Figure 6. Monocytes were cultured for 7 days with CMs from NF, CAF or MDA-MB-231 cells as well as in standard medium. All of the differentiated monocytes were then serum starved for 48 hours before obtaining the corresponding CMs. MDA-MB-231 cells were incubated with those CMs from differentiated monocytes for 24 hours. The effects of NF-, CAF- or MDA-MB-231-educated monocyte CMs on the EMT of MDA-MB-231 breast cancer cells were analysed by Western blot analysis of vimentin and E-cadherin proteins. Levels of GAPDH were used as a loading control.
